# Incidence of anxiety disorders following Parkinson’s disease: a population-based cohort study

**DOI:** 10.3389/fpsyt.2026.1788684

**Published:** 2026-05-08

**Authors:** Youngoh Bae, Jongseung Han, Sang Ryong Jeon, Wonsuk Shin, Jae June Dong, Hohyun Jung, Seung Won Lee

**Affiliations:** 1 Department of Precision Medicine, Sungkyunkwan University School of Medicine, Suwon, Republic of Korea; 2 Department of Neurological Surgery, Asan Medical Center, University of Ulsan College of Medicine, Seoul, Republic of Korea; 3 Department of Artificial Intelligence, Sungkyunkwan University, Suwon, Republic of Korea; 4 Department of Clinical Pharmacology and Therapeutics, CHA University Bundang Medical Center, Seongnam-si, Republic of Korea; 5 Department of Family Medicine, Kangbuk Samsung Hospital, Sungkyunkwan University School of Medicine, Seoul, Republic of Korea; 6 Department of Statistics, Sungshin Women’s University, Seoul, Republic of Korea; 7 Center for Data Science, Sungshin Women’s University, Seoul, Republic of Korea; 8 Human-Centered AI Institute, Sungshin Women’s University, Seoul, Republic of Korea; 9 Personalized Cancer Immunotherapy Research Center, Sungkyunkwan University School of Medicine, Suwon, Republic of Korea

**Keywords:** anxiety disorder, cohort study, incidence, Parkinson’s disease, South Korea

## Abstract

**Background:**

Parkinson’s disease is a progressive neurodegenerative disorder in which non-motor psychiatric symptoms are common. Anxiety disorder is associated with poorer clinical outcomes and reduced quality of life in Parkinson’s disease, yet population-based evidence on the risk of newly diagnosed anxiety disorder after Parkinson’s disease remains limited.

**Methods:**

We conducted a nationwide population-based matched cohort study using data from the Korean National Health Insurance Service between 2012 and 2023. Incident Parkinson’s disease was identified using diagnostic codes, and anxiety disorder was defined based on repeated clinical diagnoses. Individuals with a prior history of anxiety disorder were excluded using a three-year washout period. Patients with Parkinson’s disease were matched to general population controls at a 1:5 ratio using propensity scores. Incidence rate ratios and adjusted hazard ratios were estimated using time-stratified Cox regression models.

**Results:**

The study included 3,742 patients with Parkinson’s disease and 18,710 matched controls. The incidence rate of anxiety disorder was higher among patients with Parkinson’s disease than among controls (56.62 vs. 28.10 per 1,000 person-years), corresponding to an incidence rate ratio of 2.01. The increased risk was most pronounced during the first three years after diagnosis and again during long-term follow-up, indicating a time-dependent pattern.

**Conclusion:**

Patients with Parkinson’s disease experience a substantially higher incidence of anxiety disorder compared with the general population, with risk varying over the disease course. These findings highlight anxiety disorder as an important non-motor psychiatric manifestation of Parkinson’s disease and support the need for longitudinal mental health monitoring in this population.

## 1 Introduction

Parkinson’s disease (PD) is a representative neurodegenerative disorder characterized by progressive loss of dopaminergic neurons in the substantia nigra pars compacta, leading to cardinal motor symptoms such as resting tremor, rigidity, and bradykinesia (1). With rapid population aging, the global prevalence of PD has increased substantially, resulting in a growing healthcare and socioeconomic burden. PD is now recognized as one of the fastest-growing causes of neurological disability worldwide, highlighting the need for disease management beyond motor symptom control (2, 3).

More recently, PD has been recognized as a systemic neurodegenerative disorder that extends beyond motor manifestations to include a broad range of non-motor symptoms (4). Anxiety disorder is one of the most common psychiatric non-motor symptoms in individuals with PD (5, 6). Anxiety in PD has been associated with poorer motor and functional outcomes, accelerated cognitive and motor decline, and increased healthcare utilization (7–11). Beyond psychological reactions to chronic disability, this elevated anxiety risk is deeply rooted in PD-associated neuropathology. While PD is primarily characterized by dopaminergic depletion (12), the disease also involves extensive degeneration of serotonergic, noradrenergic, and cholinergic systems, all of which are critical for emotional regulation (13, 14). Moreover, the spread of alpha-synuclein pathology into limbic regions—including the amygdala and prefrontal cortex—directly impairs the neural circuits governing anxiety and fear response (15, 16). These neurobiological underpinnings suggest that PD patients possess an inherent vulnerability to incident anxiety disorders.

Although previous studies have shown that anxiety symptoms are common in patients with PD and have suggested possible pathophysiological links (17, 18), important limitations remain in clarifying the onset risk of anxiety disorder. Most studies have focused on prevalent symptoms rather than incident anxiety disorder after PD diagnosis, leaving post-diagnostic incidence poorly defined. In addition, analyses have largely been restricted to PD populations, limiting evidence on whether PD independently increases anxiety disorder risk compared with the general population. Anxiety risk has also been evaluated at fixed time points, preventing systematic assessment of temporal changes over the disease course. Consequently, potential heterogeneity in anxiety disorder risk across disease stages, as well as modification by age and sex, remains insufficiently characterized, and population-based evidence stratified by key demographic factors is still lacking (19).

To address these limitations, large-scale population-based longitudinal studies are needed to distinguish incident anxiety disorder after PD diagnosis and to quantify the independent contribution of PD to anxiety risk through comparison with the general population. Long-term follow-up further enables assessment of time-dependent changes in anxiety risk, while stratified analyses by age and sex allow characterization of demographic heterogeneity and identification of high-risk subgroups. Accordingly, this study evaluated the long-term and time-dependent risk of incident anxiety disorder in patients with PD compared with the general population using a nationwide cohort.

## 2 Materials and methods

### 2.1 Study design and data sources

The primary objective of this study was to investigate the independent association between Parkinson’s disease (PD) and the development of anxiety disorder, and to compare the incidence of anxiety disorder between patients with PD and the general population as a secondary objective.

Data for this study were obtained from the National Health Insurance Service claims database and the National Health Screening Program database, covering the period from January 2012 to December 2023. The study population consisted of approximately two million individuals drawn from a nationwide sample cohort, which was constructed through stratified random sampling based on age, sex, and income levels from the entire population of health insurance beneficiaries in 2012, and a retrospective cohort design was applied. South Korea operates a mandatory, universal health insurance system, and the National Health Insurance Service claims database systematically records diagnostic information based on the International Classification of Diseases, 10th Revision codes, allowing reliable estimation of disease prevalence and incidence. In addition, linkage with data from the National Health Screening Program conducted by the National Health Insurance Service enhances the accuracy and validity of the analyses (20). As participation in the NHSP is mandatory for the general population in Korea, the potential for healthy volunteer bias is minimized, and the sample is highly representative of the overall population. Although the database does not provide information on the type of diagnosing physician, diagnostic validity was enhanced by requiring repeated diagnostic records.

This study was approved by the Institutional Review Board of Sungshin Women’s University (SSWUIRB-2025-106), and the requirement for informed consent was waived due to the retrospective nature of the study.

### 2.2 Case and control groups

The case group was defined as individuals diagnosed with PD using the International Classification of Diseases, 10th Revision code G20 (**Supplementary Table 1**) during the observation period from 2015 to 2023, with confirmation of the diagnosis on at least two outpatient visits within a one-month interval. The index date was defined as the date on which PD was first identified. To include only incident cases and to minimize time-related bias and potential reverse causation, a three-year washout period from 2012 to 2014 was applied, and all individuals with a diagnosis of PD during this period were excluded (21).

Participants were further excluded if they had no health screening records within three years prior to the index date, were younger than 20 years or older than 80 years at the index date, or had a documented diagnosis of anxiety disorder within three years prior to the index date. Additional exclusion criteria including depression are detailed in **Supplementary Table 2**.

**Figure 1:**
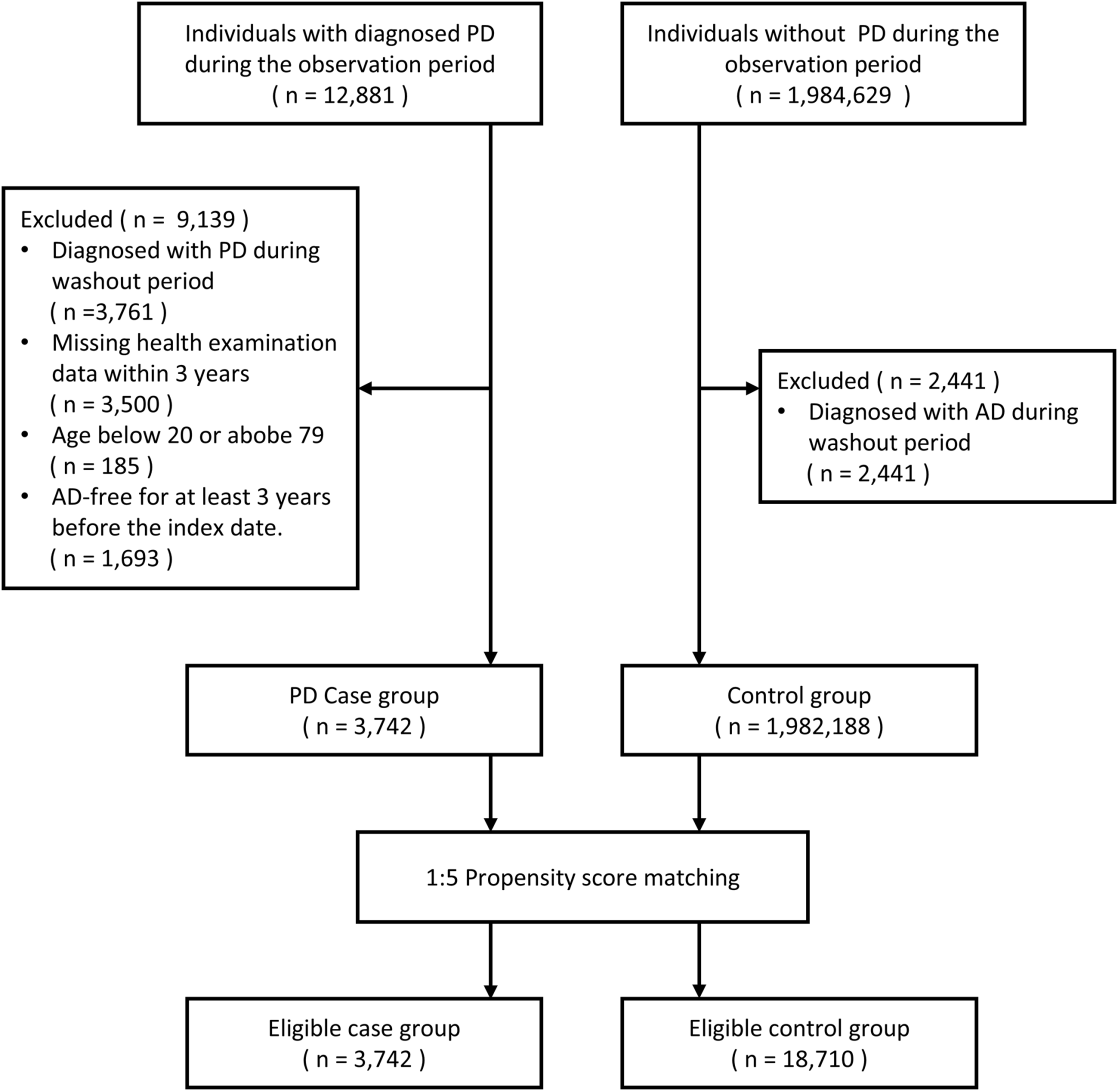
Flowchart for case and control group selection.

The control group was composed of individuals who participated in the National Health Screening Program between 2012 and 2023 and had no recorded diagnosis of PD during the same period. The same exclusion criteria applied to the case group were also applied to the control group. To minimize baseline imbalances between the PD and control groups, propensity score matching was performed at a 1:5 ratio based on sex, age, and health screening year (22). Variables included in the propensity score model were smoking status, alcohol consumption, body mass index, total cholesterol, systolic and diastolic blood pressure, fasting blood glucose, income level, triglycerides, and high-density and low-density lipoprotein cholesterol. Specific categorizations and scales for these variables are provided in **Supplementary Table 3**. For covariates with missing values, single imputation was applied (23). The process of participant selection and exclusion is illustrated in **Figure 1**.

### 2.3 Study outcome

**Figure 2:**
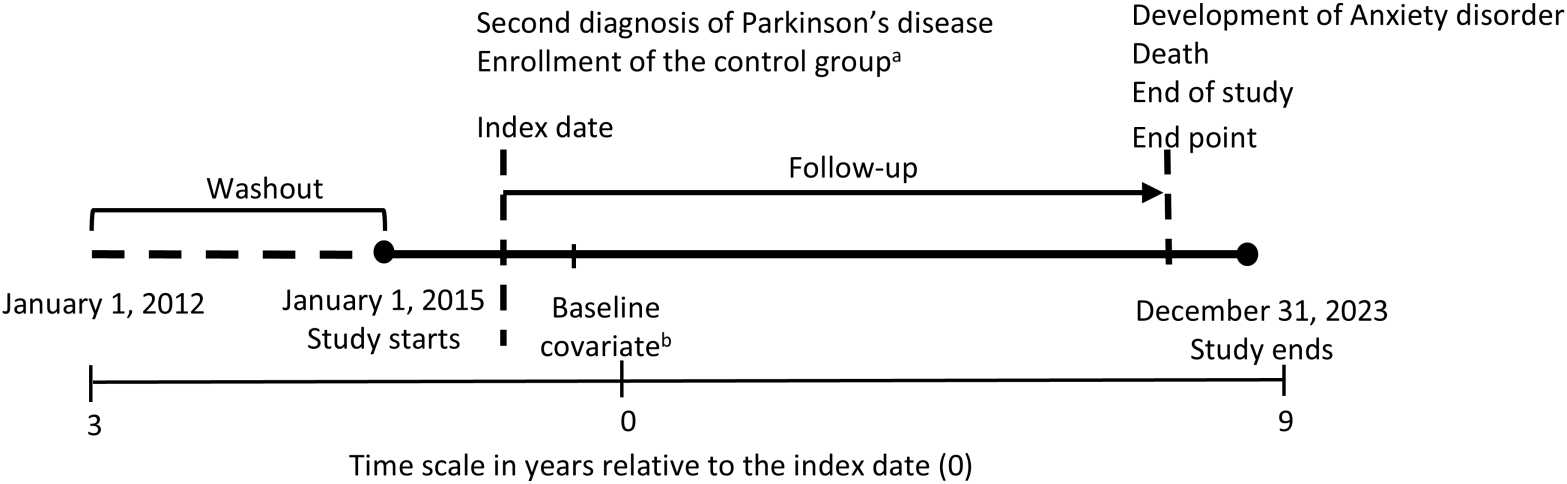
Study design and definition of baseline and follow-up. a The date of second diagnosis in matched patients with Parkinson’s disease (PD; ICD-10 G20). b Baseline covariates were assessed using data from the national health screening examination conducted closest to the index date. Follow-up started the day after the index date and ended at anxiety disorder (ICD-10 F40, F41), death, or December 31, 2023. Controls were assigned the same index date as their matched cases (matched by smoking status, alcohol consumption frequency, total cholesterol, fasting blood glucose, systolic and diastolic blood pressure, body mass index, income level, triglycerides, HDL cholesterol, and LDL cholesterol).

The primary outcome of this study was the incidence of anxiety disorder. Anxiety disorder was defined as a diagnosis of phobic anxiety disorders or other anxiety disorders (ICD-10; F40 and F41, **Supplementary Table 1**) recorded on at least two occasions (24), individuals with one diagnosis of F40 and one diagnosis of F41 were also classified as having incident anxiety disorder. The outcome index date was defined as the date of the first recorded diagnosis of anxiety disorder. For each participant, follow-up ended at the earliest occurrence of death, diagnosis of anxiety disorder, or the end of the study period (December 31, 2023), whichever came first (**Figure 2**).

### 2.4 Baseline characteristics

Baseline characteristics analyzed in this study included age, sex, smoking status, alcohol consumption, height, weight, body mass index, total cholesterol, systolic blood pressure, diastolic blood pressure, fasting blood sugar, and income level. Height and weight were treated as continuous variables, whereas age, sex, smoking status, alcohol consumption, total cholesterol, body mass index, systolic blood pressure, diastolic blood pressure, fasting blood sugar, and income level were analyzed as categorical variables. All covariates were obtained from the National Health Screening Program record closest to the index date.

### 2.5 Statistical analysis

Baseline characteristics between the case and control groups were compared using standardized differences, with a value <0.10 considered indicative of negligible imbalance. The incidence of anxiety disorder was calculated as crude incidence rates per 1,000 person-years, and comparisons between groups were performed using incidence rate ratios.

During the follow-up period, anxiety disorder–free survival probabilities were estimated using the Kaplan–Meier method, and differences between cumulative incidence curves were assessed using the log-rank test.

In the matched cohort, a primary analysis was performed using an adjusted multivariable Cox proportional hazards regression model. Parkinson’s disease status was included as the main exposure, and all covariates used in the propensity score model were simultaneously included in the model. Covariate adjustment was additionally performed in the Cox model to account for any residual imbalance after propensity score matching. Matched pairs were accounted for as strata, and reference categories for all covariates are listed in **Supplementary Table 4**. Results were presented as adjusted hazard ratios with 95% confidence intervals. The proportional hazards assumption was evaluated using scaled Schoenfeld residuals. Because the proportional hazards assumption was violated, time-stratified Cox regression models were applied to assess changes in anxiety disorder risk over time among patients with PD. The follow-up period was divided into three intervals of three years each, and hazard ratios were estimated for each interval. All statistical analyses were performed using R version 4.4.1. All tests were two-sided, and a P-value <0.05 was considered statistically significant.

### 2.6 Subgroup analysis

To assess whether the association between PD and the incidence of anxiety disorder varied according to demographic characteristics, additional exploratory subgroup analyses were conducted stratified by age and sex. Within each subgroup, Cox proportional hazards regression models were applied to evaluate the association between PD and the risk of incident anxiety disorder. Variables used for stratification were excluded from the corresponding regression models.

## 3 Results

### 3.1 Study population

According to the predefined inclusion and exclusion criteria, a total of 3,742 patients with PD and 18,710 control individuals were included in the final analysis (**Figure 1**). The mean follow-up duration was 3.04 years (SD, 2.35) in the PD case group and 3.67 years (SD, 2.48) in the control group. The maximum follow-up period spanned nine years, from 2015 to 2023.

### 3.2 Baseline characteristics

Baseline demographic and clinical characteristics of the PD and control groups after propensity score matching are summarized in **Table 1**. Comparison of baseline characteristics using standardized differences indicated that most covariates had values below 0.10, suggesting minimal imbalance between the two groups, although a modest residual imbalance was observed for alcohol consumption.

**Table 1:** Baseline demographic characteristics of patients with Parkinson’s disease and control group.

Characteristics		Case group (n =3,724) (%)	Control group (n = 18,710) (%)	Standardized difference
Age (years)	20–29	11 (0.29%)	55 (0.29%)	0.00
	30–39	79 (2.11%)	395 (2.11%)	
	40–49	265 (7.08%)	1325 (7.08%)	
	50–59	817 (21.83%)	4085 (21.83%)	
	60–69	1362 (36.40%)	6810 (36.40%)	
	≥70	1208 (32.28%)	6040 (32.28%)	
Sex	Male	1848 (49.39%)	9240 (49.39%)	0.00
	Female	1894 (50.61%)	9470 (50.61%)	
Smoking status	Yes	288 (7.70%)	1347 (7.20%)	0.04
	No	2713 (72.50%)	13426 (71.76%)	
	Ex-Smoking	739 (19.75%)	3923 (20.97%)	
Frequency of alcohol consumption (per week)	0	1677 (44.82%)	8575 (45.83%)	0.07
	1–2	694 (18.55%)	4115 (21.99%)	
	≥3	177 (4.73%)	962 (5.14%)	
Weight (kg, mean ± SD)		61.59 ± 10.76	61.77 ± 10.56	0.02
Height (cm, mean ± SD)		159.10 ± 9.24	159.33 ± 9.07	0.02
BMI (kg/m^2^)	<18.5	111 (2.97%)	469 (2.51%)	0.06
	18.5 to <25	2145 (57.32%)	11097 (59.31%)	
	≥25	1484 (39.66%)	7137 (38.15%)	
Total cholesterol (mg/dL)	<200	1678 (44.84%)	7998 (42.75%)	0.06
	≥200	838 (22.39%)	4183 (22.36%)	
Systolic Blood Pressure (mmHg)	<120	986 (26.35%)	4980 (26.62%)	0.01
	120 to <140	1867 (49.89%)	9408 (50.28%)	
	≥140	808 (21.59%)	4050 (21.65%)	
Diastolic Blood Pressure (mmHg)	<80	2216 (59.22%)	11168 (59.69%)	0.06
	80 to <90	1081 (28.89%)	5540 (29.61%)	
	≥90	364 (9.73%)	1730 (9.25%)	
FBS (mg/dL)	<100	1657 (44.28%)	8485 (45.35%)	0.09
	100 to <126	1400 (37.41%)	7298 (39.01%)	
	≥126	604 (16.14%)	2652 (14.17%)	
Income	Low	1342 (35.86%)	6648 (35.53%)	0.04
	High	2302 (61.52%)	11437 (61.13%)	

BMI, body mass index; FBS, fasting blood sugar; SD, standard deviation.

### 3.3 Incidence of anxiety disorder

During the entire follow-up period, 644 individuals in the PD case group were newly diagnosed with anxiety disorder, corresponding to an incidence rate of 56.62 per 1,000 person-years (95% CI, 52.31–61.02). In contrast, 1,930 individuals in the control group were diagnosed with anxiety disorder, yielding an incidence rate of 28.10 per 1,000 person-years (95% CI, 26.85–29.37). Accordingly, the incidence rate ratio for anxiety disorder among patients with PD was 2.01 (95% CI, 1.84–2.20), indicating approximately a twofold higher incidence compared with the control group (**Table 2**).

**Table 2:** Crude incidence rate and incidence rate ratio of anxiety disorders in PD.

Characteristics		Case group (n = 3,742)			Reference group (n = 18,710)			IRR (95% CI)
		Cases	Person-years	IR per 1000 person-years (95% CI)	Cases	Person-years	IR per 1000 person-years (95% CI)	
All		644	11373.51	56.62 (52.31–61.02)	1930	68674.96	28.10 (26.85–29.37)	2.01 (1.84–2.20)
Age (years)	<60	205	3513.57	58.35 (50.38–66.60)	417	20549.35	20.29 (18.35–22.24)	2.88 (2.43–3.40)
	≥60	439	7859.94	55.85 (50.64–61.20)	1513	48125.62	31.44 (29.86–33.04)	1.78 (1.60–1.98)
Sex	Male	263	5484.46	47.95 (42.30–53.79)	753	33781.31	22.29 (20.72–23.89)	2.15 (1.87–2.48)
	Female	381	5889.05	64.70 (58.24–71.32)	1177	34893.65	33.73 (31.81–35.68)	1.92 (1.71–2.15)
Sex & Age (years)	Male, <60	89	1790.5	49.71 (39.65–60.32)	153	10450.86	14.64 (12.34–17.03)	3.40 (2.61–4.41)
	Male, ≥60	174	3693.96	47.10 (40.34–54.14)	600	23330.45	25.72 (23.66–27.77)	1.83 (1.55–2.17)
	Female, <60	116	1723.06	67.32 (55.13–80.09)	264	10098.49	26.14 (23.07–29.31)	2.58 (2.07–3.20)
	Female, ≥60	265	4165.98	63.61 (56.17–71.29)	913	24795.17	36.82 (34.44–39.24)	1.73 (1.51–1.98)
Smoking status	Yes	43	846.31	50.81 (36.63–66.17)	108	5080.36	21.26 (17.32–25.39)	2.39 (1.68–3.40)
	No	490	8250.71	59.39 (54.18–64.72)	1515	49133.63	30.83 (29.29–32.40)	1.93 (1.74–2.13)
	Ex-Smoking	110	2271.85	48.42 (39.62–57.66)	306	14407.79	21.24 (18.88–23.67)	2.28 (1.83–2.83)
Frequency of alcohol consumption (per week)	0	208	3705.73	56.13 (48.57–63.96)	648	22900.62	28.30 (26.16–30.48)	1.98 (1.70–2.32)
	1–2	102	1762.59	57.87 (47.09–69.22)	271	12776.01	21.21 (18.71–23.79)	2.73 (2.17–3.43)
	≥3	21	539.5	38.92 (24.10–55.61)	82	3374.09	24.30 (19.26–29.64)	1.60 (0.99–2.59)
BMI (kg/m^2^)	<18.5	14	318.25	43.99 (22.00–69.13)	43	1600.73	26.86 (19.37–34.98)	1.64 (0.90–2.99)
	18.5 to <25	391	6355.59	61.52 (55.54–67.66)	1179	41110.51	28.68 (27.05–30.33)	2.15 (1.91–2.41)
	≥25	239	4699.04	50.86 (44.48–57.46)	708	25934.8	27.30 (25.29–29.34)	1.86 (1.61–2.16)
Total cholesterol (mg/dL)	<200	308	5689.96	54.13 (48.16–60.28)	1001	33747.01	29.66 (27.82–31.50)	1.82 (1.61–2.07)
	≥200	183	3160.77	57.90 (49.67–66.44)	511	19302.81	26.47 (24.19–28.80)	2.19 (1.85–2.59)
Income	Low	226	4067.86	55.56 (48.43–62.93)	666	24117.94	27.61 (25.54–29.73)	2.01 (1.73–2.34)
	High	394	7041.41	55.95 (50.56–61.49)	1193	42235.41	28.25 (26.66–29.86)	1.98 (1.77–2.22)

IR, incidence rate; IRR, incidence rate ratio; CI, confidence interval; PD, Parkinson’s disease.

In the age-stratified analysis, among individuals younger than 60 years, the incidence rate of anxiety disorder in the PD case group was 58.35 per 1,000 person-years (95% CI, 50.38–66.60), which was approximately 2.88 times higher than that of the control group at 20.29 per 1,000 person-years (95% CI, 18.35–22.24), with an incidence rate ratio of 2.88 (95% CI, 2.43–3.40). Among individuals aged 60 years or older, the incidence rate of anxiety disorder in the PD case group was 55.85 per 1,000 person-years (95% CI, 50.64–61.20), which was approximately 1.78 times higher than that of the control group at 31.44 per 1,000 person-years (95% CI, 29.86–33.04), corresponding to an incidence rate ratio of 1.78 (95% CI, 1.60–1.98).

In the sex-stratified analysis, among male patients with PD, the incidence rate of anxiety disorder was 47.95 per 1,000 person-years (95% CI, 42.30–53.79), which was approximately 2.15 times higher than that of the control group at 22.29 per 1,000 person-years (95% CI, 20.72–23.89), with an incidence rate ratio of 2.15 (95% CI, 1.87–2.48). Among female participants, the incidence rate of anxiety disorder in the PD case group was 64.70 per 1,000 person-years (95% CI, 58.24–71.32), which was approximately 1.92 times higher than that of the control group at 33.73 per 1,000 person-years (95% CI, 31.81–35.68), corresponding to an incidence rate ratio of 1.92 (95% CI, 1.71–2.15).

**Figure 3:**
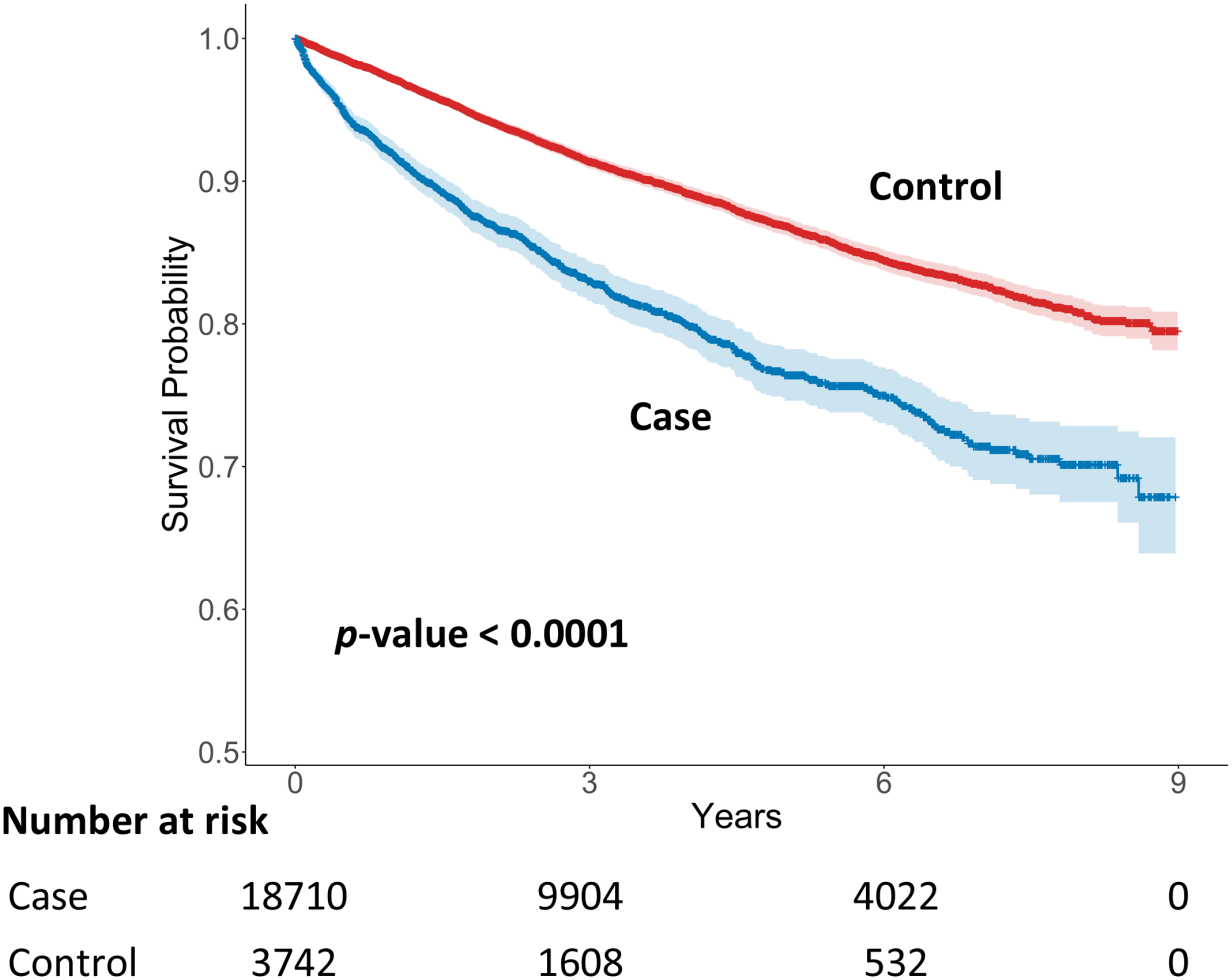
Kaplan–Meier survival curves for psychiatric disorder following Parkinson’s disease diagnosis.

Kaplan–Meier survival analysis demonstrated a significantly lower anxiety disorder–free survival probability in patients with Parkinson’s disease compared with matched controls throughout the follow-up period (log-rank test, p < 0.001; **Figure 3**). Complementary log-log plots (**Supplementary Figure 1**) showed non-linear patterns over time, supporting the use of Cox models without parametric assumptions.

### 3.4 Subgroup analysis

**Figure 4:**
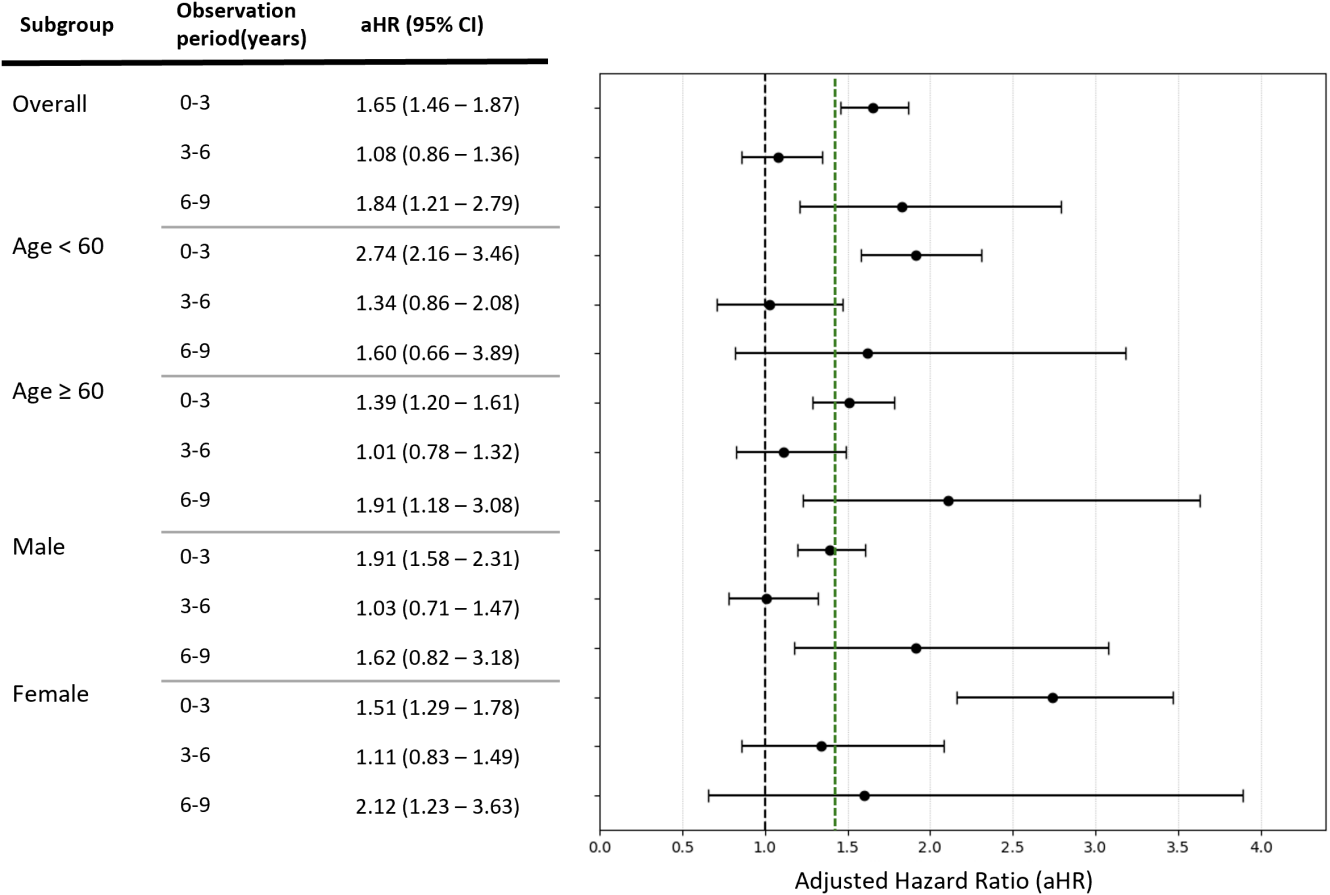
Adjusted hazard ratios for incident Anxiety disorder following Parkinson’s disease. Green dashed line indicates the average adjusted hazard ratio across all subgroups. CI, confidence interval; aHR, adjusted hazard ratio.

In the time-stratified Cox regression analysis, the risk of incident anxiety disorder among patients with PD was most pronounced during the early period following diagnosis (0–3 years), with an adjusted hazard ratio of 1.65 (95% CI, 1.46–1.87). During the intermediate follow-up period (3–6 years), the increase in risk was temporarily attenuated. However, a significant increase in risk was observed again during the long-term follow-up period (6–9 years), with an adjusted hazard ratio of 1.83 (95% CI, 1.21–2.79). Notably, the early increase in risk was most evident among patients younger than 60 years and among male patients, indicating that the risk of anxiety disorder was highest shortly after PD diagnosis in younger individuals and men (**Figure 4**).

Additionally, in the analysis of metabolic profiles, a relatively higher risk of incident anxiety disorder was observed among individuals with total cholesterol levels of 200 mg/dL or higher compared to those with lower levels.

## 4 Discussion

### 4.1 Principal findings

Using a nationwide Korean National Health Insurance Service cohort comprising approximately 2 million individuals followed from 2012 to 2023, this study compared the risk of incident anxiety disorders among patients with Parkinson’s disease(PD) and matched control subjects. Patients with PD exhibited an approximately twofold higher risk of developing anxiety disorders compared with controls, and this association remained robust after adjustment for major demographic and clinical confounding factors. The risk of incident anxiety disorders was not uniform over time following the diagnosis of PD. Instead, a time-dependent pattern was observed, with relatively higher risks during the early post-diagnosis period and again during long-term follow-up. In addition, the relative risk of anxiety disorders was more pronounced among patients younger than 60 years and among male patients.

### 4.2 Comparison with previous studies

Several previous cohort studies have reported an increased risk of anxiety symptoms and anxiety disorders among patients with PD. A hospital-based longitudinal cohort study conducted in the Netherlands followed 409 patients with PD for a mean duration of 2.6 years and found that approximately 19% of patients without anxiety at the time of diagnosis developed new-onset anxiety symptoms during follow-up (25). This study demonstrated that anxiety may newly emerge relatively frequently in patients with PD even over a short follow-up period, which is generally consistent with our findings showing a significantly higher risk of incident anxiety disorders in patients with PD compared with control subjects. However, the prior study was limited to a hospital-based cohort and did not include a general population control group, nor did it quantify the relative risk of anxiety disorders in the form of hazard ratios or other comparative risk estimates.

Another hospital-based prospective cohort study conducted in the United States followed 105 patients with PD for approximately 3 years and examined the occurrence of anxiety symptoms (26). In that study, about 57% of patients experienced clinically significant levels of anxiety symptoms during the follow-up period, suggesting that anxiety symptoms commonly accompany PD over the disease course. However, this study was likewise limited to a small, hospital-based cohort, had a relatively short follow-up period, and focused on descriptive analyses of the presence and temporal changes of anxiety symptoms, without quantitatively assessing the risk of incident anxiety disorders in comparison with a general population control group.

Concerning the timing of anxiety onset, previous studies have reported that anxiety may be observed both before and after the diagnosis of PD. Some studies have shown that anxiety may occur before PD diagnosis, while in many patients it develops after diagnosis during follow-up (27). However, these studies largely focused on describing the presence or timing of anxiety onset and did not systematically evaluate how the risk of incident anxiety disorders changes according to time elapsed since PD diagnosis.

In contrast, the present study directly compared the risk of incident anxiety disorders between patients with PD and the general population using a nationwide, population-based cohort of approximately 2 million individuals. By applying time-stratified analyses over a follow-up period of up to 9 years, we systematically evaluated changes in the risk of anxiety disorders according to time elapsed since PD diagnosis. This approach addresses the limitations of prior small, hospital-based cohort studies that primarily focused on the presence or timing of anxiety symptoms. It also extends previous research by demonstrating, at the population level, that anxiety disorders represent an important non-motor feature of PD, with risk patterns that vary dynamically across the disease course.

### 4.3 Time-dependent risk

The time-dependent risk pattern observed in this study indicates that the risk of incident anxiety disorders in patients with PD varies over the disease course rather than remaining constant after diagnosis. The increased risk observed both shortly after diagnosis and during long-term follow-up suggests that anxiety disorders are not solely attributable to surveillance effects or transient post-diagnostic reactions. Instead, anxiety risk may re-emerge as the disease progresses. While early risk increases may relate to clinical and environmental changes following diagnosis, later increases may be associated with cumulative non-motor symptom burden and ongoing neurodegenerative processes. These findings indicate that anxiety disorders require repeated assessment throughout the course of PD rather than a single evaluation after diagnosis.

### 4.4 Age & sex

The pronounced risk of incident anxiety disorders among younger patients with PD may reflect the significant psychological burden associated with the disease’s onset during critical life stages. For individuals younger than 60 years, a PD diagnosis often coincides with peak periods of employment, social engagement, and family responsibilities, thereby imposing greater stress compared to older populations (19, 28). Furthermore, in younger patients, disease-related neurodegenerative processes may contribute more directly to non-motor symptoms than age-related effects alone (29), suggesting that the impact of PD on anxiety risk is modulated less by chronological age than by the additional burden imposed by the disease itself.

In sex-stratified analyses, the relatively higher risk observed in male patients with PD suggests that the incremental effect of PD on anxiety risk may be more substantial in men, especially since the baseline prevalence of anxiety is generally higher among women in the general population. This aligns with previous reports highlighting sex-specific patterns in the clinical expression of non-motor symptoms in PD (30). However, these interpretations should be made with caution, as multiple testing adjustments were not performed and the possibility of type I error cannot be excluded.

### 4.5 Clinical & public health implications

The findings of this study suggest that systematic screening and assessment for anxiety disorders are warranted from the early stages after PD diagnosis. Given the relatively higher risk observed among younger and male patients, proactive monitoring strategies targeting these high-risk groups may be particularly important in clinical practice. Anxiety disorders in patients with PD have been shown to be closely associated with falls, hospitalizations, poorer prognosis, and increased mortality (31, 32). Accordingly, early psychiatric intervention following PD diagnosis may improve quality of life and help reduce long-term healthcare utilization, costs, and complication burden. Overall, the present study provides clinical evidence supporting the need to move beyond a motor symptom–centered approach toward an integrated management strategy that incorporates mental health in the care of patients with PD.

### 4.6 Strengths

The present study analyzed the association between PD and anxiety disorders using population-based data that combined nationwide health insurance claims and health screening records. Only incident cases of both PD and anxiety disorders were included, with the application of a washout period to minimize the influence of pre-existing conditions. A follow-up period of up to 9 years allowed for the evaluation of not only short-term risk but also long-term changes in risk over time. In addition, propensity score matching and time-stratified Cox regression models were applied to account for demographic and clinical confounding factors and to precisely capture temporal variations in risk.

### 4.7 Limitations and future directions

The findings of this study should be interpreted in light of several limitations.

First, as this study was based on health insurance claims and health screening data, anxiety disorders were identified using ICD-10 diagnostic codes and healthcare utilization records. Accordingly, mild or PD-specific anxiety may not have been fully captured, and diagnostic misclassification cannot be entirely excluded (33). In addition, although individuals with baseline depression were excluded and a three-year washout period was applied to minimize prevalent cases, which is longer than in prior claims-based studies, residual confounding due to subclinical symptoms may remain, and pre-existing conditions cannot be fully excluded.

Second, the potential for residual confounding remains. Due to the inherent characteristics of the database, detailed clinical information on PD, including disease severity, clinical subtypes, and specific non-motor burdens like sleep disturbances, could not be adequately reflected. Because previous longitudinal studies identify these specific clinical features as strong independent predictors of anxiety in PD (26), the inability to fully adjust for them restricts a more refined assessment of heterogeneity in anxiety disorder risk.

Third, information on the use of antiparkinsonian medications, anxiolytics, and antidepressants was limited, precluding a comprehensive analysis accounting for treatment patterns. As medication exposure may affect anxiety risk, residual confounding related to unmeasured treatment effects remains possible.

Fourth, generalizability may be limited by the specific healthcare system and ethnic homogeneity of the cohort. Because the baseline prevalence and phenotypic presentation of non-motor symptoms like anxiety can vary significantly between Asian and Western populations (34), caution is required when extrapolating these findings to other ethnic backgrounds.

Fifth, surveillance bias should be considered. PD patients require more frequent healthcare interactions, with a previous study reporting higher annual outpatient visits in PD patients compared to controls (28.9 vs. 23.4 visits/year) (35). This increased healthcare utilization creates more opportunities for anxiety diagnosis, potentially overestimating its incidence in the PD group.

Sixth, death was not considered as a competing risk. Previous evidence indicates that the 10-year mortality rate for Korean PD patients is substantially higher than that of controls (47.9% vs. 20.3%; HR = 2.96) (36). Given this elevated mortality, death may act as a competing event, potentially leading to an overestimation of the incident anxiety risk in the PD group.

Considering these limitations, the associations observed in this study should not be interpreted as causal, given the observational nature of the design. Future studies using more detailed data that incorporate clinical severity, non-motor symptom burden, and medication use are warranted to further examine the temporal and individual-level heterogeneity in the risk of anxiety disorders following PD.

### 4.8 Conclusion

Using a nationwide population-based cohort, this study demonstrated that the risk of incident anxiety disorder is significantly higher among patients with PD compared with the general population. This increased risk was particularly evident during the early period after diagnosis and during long-term follow-up, as well as among younger patients and male patients. These findings indicate that anxiety disorder represents an important non-motor manifestation of PD that warrants ongoing attention throughout the disease course and underscore the need for early screening and targeted intervention strategies focused on high-risk groups from the time of diagnosis.

## Data Availability

The data analyzed in this study is subject to the following licenses/restrictions: The dataset analyzed in this study is not publicly available due to legal and ethical restrictions imposed by the Korean National Health Insurance Service. Access to the data is permitted only to approved researchers under data use agreements and cannot be shared publicly. Requests to access these datasets should be directed to Hohyun Jung, hhjung@sungshin.ac.kr.
