# Supplementary material for: Incidence of anxiety disorders following Parkinson’s disease: a population-based cohort study
Source: Front Psychiatry. 2026 May 8;17:1788684. doi: 10.3389/fpsyt.2026.1788684 (PMC13194130; doi:10.3389/fpsyt.2026.1788684)
Supplement: Supplementary file 1 [file Table1.docx]

Supplementary Material

# Supplementary Tables

**Supplementary Table S1.** International Classification of Diseases, 10th Revision (ICD-10)

| **Disease** | **ICD-10 code** |
| --- | --- |
| Exposure disease | |
| Parkinson’s disease | G20 |
| Outcome disease | |
| Anxiety disorder | F40, F41 |

**Supplementary Table S2.** International Classification of Diseases, 10th Revision (ICD-10) or operation codes used for exclusion criteria in study cohort selection

| **Disease** | **ICD-10 code** |
| --- | --- |
| **Exposure** | |
| Parkinson’s disease | G20 |
| Outcome (washout prevalence exclusion) | |
| Anxiety disorder | F40, F41 |
| Parkinsonism-related diagnoses (exclusion for non-idiopathic/secondary parkinsonism) | |
| Secondary parkinsonism | G21 |
| Parkinsonism in diseases classified elsewhere | G22 |
| Psychiatric disorders (major exclusions) | |
| Schizophrenia, schizotypal and delusional disorders | F20–F29 |
| Bipolar disorder and manic episode | F30–F31 |
| Depressive episode and recurrent depressive disorder | F32–F33 |
| Major neurologic disorders (major exclusions) | |
| Dementia | F00–F03 |
| Stroke | I60–I63 |
| Multiple sclerosis | G35 |
| Other degenerative diseases of the nervous system | G10–G14 |
| Specific sleep disorders (pre-existing; exclusion) | |
| REM sleep behavior disorder | G47.2 |
| Sleep apnea | G47.3 |
| Narcolepsy | G47.4 |

**Supplementary Table S3.** Logistic regression model for estimating propensity scores

| **Variable** | **Categories** |
| --- | --- |
| Income level | Low / High |
| Smoking status | Never / Former / Current |
| Alcohol consumption | 0 / 1–2 / ≥3 (times/week) |
| Body mass index | <18.5 / 18.5–<25 / ≥25 |
| Total cholesterol | <200 / ≥200 (mg/dL) |
| Systolic BP | <120 / 120–<140 / ≥140 (mmHg) |
| Diastolic BP | <80 / 80–<90 / ≥90 (mmHg) |
| Fasting blood glucose | <100 / 100–<126 / ≥126 (mg/dL) |
| Triglycerides | <150 / ≥150 (mg/dL) |
| HDL cholesterol | Normal / Low (male: <40mg/dL / female: 50 mg/dL) |
| LDL cholesterol | <130 / ≥130 mg/dL |

**Supplementary Table S4.** Reference categories for categorical covariates included in the Cox proportional hazards model.

| **Variable** | **Categories** |
| --- | --- |
| Age | Continuous (per 1-year increase) |
| BMI | Continuous |
| Sex | Male |
| Smoking | Ex-smoking |
| Alcohol consumption | 0 (times/week) |
| Total cholesterol | Low (<200 mg/dL) |
| Income | Low |

**Supplementary Figure S1.** Complementary log-log transformed survival curves for Parkinson’s disease and matched controls.

**
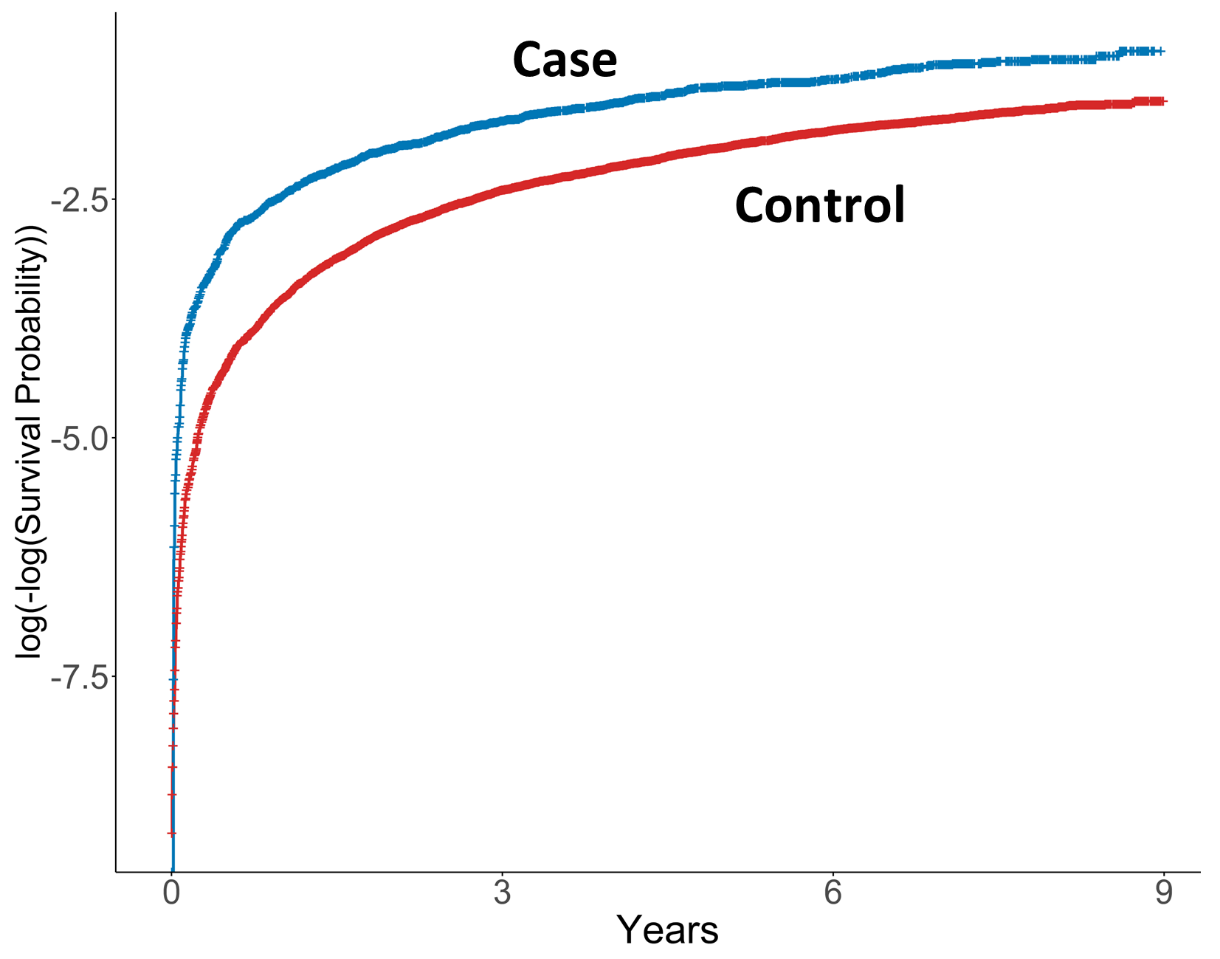
**
